# Presentation cardiac troponin and early computed tomography coronary angiography in patients with suspected acute coronary syndrome: a pre-specified secondary analysis of the RAPID-CTCA trial

**DOI:** 10.1093/ehjacc/zuac057

**Published:** 2022-06-01

**Authors:** Kang Ling Wang, Carl Roobottom, Jason E Smith, Steve Goodacre, Katherine Oatey, Rachel O’Brien, Robert F Storey, Nick Curzen, Liza Keating, Attila Kardos, Dirk Felmeden, Praveen Thokala, Nicholas L Mills, David E Newby, Alasdair J Gray

**Affiliations:** Centre for Cardiovascular Science, University of Edinburgh, Chancellor’s Building, 49 Little France Crescent, Edinburgh EH16 4SB, UK; School of Medicine, National Yang Ming Chiao Tung University, Taipei, Taiwan; General Clinical Research Center, Taipei Veterans General Hospital, Taipei, Taiwan; Department of Radiology, University Hospitals Plymouth NHS Trust, Plymouth, UK; Emergency Department, University Hospitals Plymouth NHS Trust, Plymouth, UK; School of Health and Related Research, University of Sheffield, Sheffield, UK; Edinburgh Clinical Trials Unit, University of Edinburgh, Edinburgh, UK; Department of Emergency Medicine, Royal Infirmary of Edinburgh, Edinburgh, UK; Department of Infection, Immunity and Cardiovascular Disease, University of Sheffield, Sheffield, UK; Faculty of Medicine, University of Southampton, Southampton, UK; Department of Cardiology, University Hospital Southampton NHS Foundation Trust, Southampton, UK; Department of Emergency Medicine, Royal Berkshire NHS Foundation Trust, Reading, UK; Translational Cardiovascular Research Group, Milton Keynes University Hospital NHS Foundation Trust, Milton Keynes, UK; Faculty of Medicine and Health Science, University of Buckingham, Buckingham, UK; Department of Cardiology, Torbay and South Devon NHS Foundation Trust, Torquay, UK; School of Health and Related Research, University of Sheffield, Sheffield, UK; Centre for Cardiovascular Science, University of Edinburgh, Chancellor’s Building, 49 Little France Crescent, Edinburgh EH16 4SB, UK; Usher Institute, University of Edinburgh, Edinburgh, UK; Centre for Cardiovascular Science, University of Edinburgh, Chancellor’s Building, 49 Little France Crescent, Edinburgh EH16 4SB, UK; Department of Emergency Medicine, Royal Infirmary of Edinburgh, Edinburgh, UK; Usher Institute, University of Edinburgh, Edinburgh, UK

**Keywords:** Acute coronary syndrome, Cardiac troponin, Computed tomography coronary angiography, Non-invasive testing

## Abstract

**Aims:**

To evaluate the potential associations between presentation cardiac troponin and the clinical impact of early computed tomography coronary angiography (CTCA) in intermediate-risk patients with suspected acute coronary syndrome.

**Methods and results:**

In a large multicentre randomized controlled trial of patients with intermediate-risk chest pain due to suspected acute coronary syndrome, early CTCA had no effect on the primary outcome—death or subsequent Type 1 or 4b myocardial infarction—but reduced the rate of invasive coronary angiography. In this pre-specified secondary analysis, cardiovascular testing and clinical outcomes were compared between those with or without cardiac troponin elevation at presentation. Of 1748 patients, 1004 (57%) had an elevated cardiac troponin concentration and 744 (43%) had a normal concentration. Patients with cardiac troponin elevation had a higher Global Registry of Acute Coronary Events score (132 vs. 91; *P* < 0.001) and were more likely to have obstructive coronary artery disease (59 vs. 33%; *P* < 0.001), non-invasive (72 vs. 52%; *P* < 0.001) and invasive (72 vs. 38%; *P* < 0.001) testing, coronary revascularization (47 vs. 15%; *P* < 0.001), and the primary outcome (8 vs. 3%; *P* = 0.007) at 1 year. However, there was no evidence that presentation cardiac troponin was associated with the relative effects of early CTCA on rates of non-invasive (*P*_interaction_ = 0.33) and invasive (*P*_interaction_ = 0.99) testing, coronary revascularization (*P*_interaction_ = 0.57), or the primary outcome (*P*_interaction_ = 0.41).

**Conclusion:**

Presentation cardiac troponin had no demonstrable associations between the effects of early CTCA on reductions in non-invasive and invasive testing, or the lack of effect on coronary revascularization or the primary outcome in intermediate-risk patients with suspected acute coronary syndrome.

## Graphical Abstract

**Graphical Abstract:**
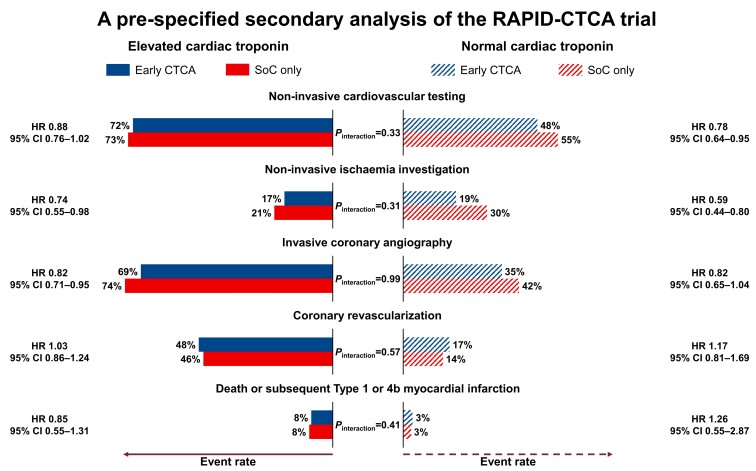
Early computed tomography coronary angiography in suspected acute coronary syndrome with or without cardiac troponin elevation.

## Introduction

Cardiac troponin is a highly specific marker of myocardial injury that is central to the diagnosis of myocardial infarction in patients with acute chest pain as well as being a key determinant of prognosis.^1^ However, with the advent of high-sensitivity cardiac troponin testing, increasing numbers of patients are being identified with cardiac troponin elevation above the 99th centile upper reference limit, which may reflect acute or chronic myocardial injury or Type 2 myocardial infarction rather than Type 1 myocardial infarction.^2^ Indeed, 1 in 20 patients presenting to the hospital and undergoing a blood test for any reason had a high-sensitivity cardiac troponin concentration above the 99th centile upper reference limit.^3^ This distinction is important, because evidence-based pharmacological and mechanical interventions that are used to treat Type 1 myocardial infarction have not yet been shown to benefit those with myocardial injury or Type 2 myocardial infarction, and may even result in harm to such patients.^4,5^ In fact, 30% of patients undergoing invasive coronary angiography with a provisional diagnosis of non-ST-segment elevation myocardial infarction are found to have no coronary artery stenosis.^6^

Patients with acute chest pain and no cardiac troponin elevation represent a heterogenous group, with a diagnosis ranging from stable or unstable angina to non-ischaemic cardiac pathologies or non-cardiac causes.^7^ The distinction between these patient groups is also critical since the former will potentially benefit from dual antiplatelet therapy and coronary revascularization, and the latter may receive such therapy and invasive testing inappropriately. Consequently, early non-invasive anatomical assessment for the presence of coronary artery disease could avoid unnecessary investigations in those without coronary artery disease and conversely, potentially improve treatment and outcomes in those with unrecognized disease.^8,9^

The Rapid Assessment of Potential Ischaemic Heart Disease with CTCA (RAPID-CTCA) trial recently reported that, among intermediate-risk patients with suspected acute coronary syndrome, the rates of invasive coronary angiography and other non-invasive investigations for coronary artery disease and myocardial ischaemia were reduced with early computed tomography coronary angiography (CTCA), although this did not impact on the rates of coronary revascularization or the primary outcome—death or subsequent Type 1 or 4b myocardial infarction at 1 year.^10^ In this pre-specified secondary analysis, we aimed to evaluate the relationship between presentation cardiac troponin and clinical effects of early CTCA in intermediate-risk patients with suspected acute coronary syndrome.

## Methods

### Patients

The RAPID-CTCA trial (ClinicalTrials.gov identifier, NCT02284191) enrolled 1749 patients, with 1748 available for analysis, with suspected acute coronary syndrome at 37 sites across the UK between March 2015 and June 2019.^10^ Eligible patients had one or more of following criteria: (i) prior coronary artery disease; (ii) electrocardiographic abnormalities; or (iii) an elevated cardiac troponin concentration. Those with any high-risk features (such as ST-segment elevation or ST-segment depression >3 mm on the electrocardiogram), inability to undergo CTCA, or either known obstructive coronary artery disease (within 2 years) or normal coronary arteries (within 5 years) by invasive coronary angiography or CTCA were excluded. Patients were randomized 1:1, grouped by site, and with variable block sizes (4–8), to standard of care plus early CTCA or standard of care only. The trial was conducted with the approval of the South East Scotland Research Ethics Committee, and all patients provided written informed consent.

### Cardiac troponin

Cardiac troponin concentrations were measured using a high-sensitivity or contemporary assay in local accredited clinical biochemistry laboratories according to standard clinical practice at each site. Assays varied between sites, and local laboratory reference standards, based upon the recommended 99th centile upper reference limit, were used to define the threshold for cardiac troponin elevation.

### Study outcomes

Data on the use of non-invasive imaging were systematically collected in the RAPID-CTCA trial. For the purposes of this substudy, non-invasive cardiovascular testing was *post hoc* categorized into either ischaemia (exercise electrocardiogram, stress echocardiogram, stress cardiac magnetic resonance imaging or angiogram, stress nuclear myocardial perfusion imaging, or CTCA other than trial assignment) or other cardiac (echocardiogram, cardiac magnetic resonance imaging, or electrocardiographic rhythm monitoring) investigations.

Invasive coronary angiography and coronary revascularization were the secondary outcomes in the RAPID-CTCA trial, whereas the primary outcome of the trial was death or subsequent Type 1 or 4b myocardial infarction adjudicated by an independent clinical endpoint committee blinded to trial assignment. Myocardial infarction was defined according to the Third Universal Definition.^11^

### Statistical analysis

In this pre-specified secondary analysis, patients were categorized into two subgroups according to their presentation cardiac troponin concentration (above or below the 99th centile upper reference limit). The initial cardiac troponin concentration was not available in three patients, for whom the second cardiac troponin concentration was used.

Demographics and clinical characteristics were summarized by cardiac troponin concentration with medians (interquartile ranges) for continuous variables and frequencies (percentages) for categorical variables, and differences were assessed with the Mann–Whitney *U* test and the χ^2^ test as appropriate. Outcomes were presented graphically with Kaplan–Meier estimates of cumulative incidences. Cox and logistic models were used to test the interaction between trial assignment and subgroups and to derive effect estimates and their 95% confidence intervals for patients with an elevated or a normal cardiac troponin concentration. Models were adjusted for fixed [Global Registry of Acute Coronary Events (GRACE) score (using a restricted cubic spline) and prior coronary artery disease] and random effects (study site).^12,13^ To examine the temporal effects of early CTCA on the rate of invasive coronary angiography, a landmark analysis was performed. A 30-day landmark was chosen because the first few weeks after acute coronary syndrome are a high-risk period for adverse outcomes,^14^ and prior studies suggested that early CTCA was associated with an early rise in the rate of invasive coronary angiography.^15^*Post hoc* sensitivity analyses were conducted by including data only from sites using a high-sensitivity cardiac troponin assay since trial inception as well as exploring whether patients with an elevated cardiac troponin concentration who were discharged without a diagnosis of myocardial infarction differed from those who had a diagnosis of myocardial infarction. Because this study was exploratory, there was no adjustment for multiplicity.

All analyses were presented on an intention-to-treat basis and were conducted using SAS version 9.4 (SAS Institute, Cary, NC, USA).

## Results

### Baseline characteristics

Of 37 sites, 28 used a high-sensitivity cardiac troponin I or T assay since trial inception, two adopted a high-sensitivity assay during the trial, six remained with a contemporary assay, and one used both a contemporary point of care and a high-sensitivity laboratory assay (see Supplementary material online, *Table S1*).

Among 1748 patients, 1004 (57%) had an elevated and 744 (43%) had a normal cardiac troponin concentration at presentation (*Table 1*). Patients with an elevated cardiac troponin concentration were of similar age but more likely to be men (67 vs. 59%; *P* < 0.001), having a former or current smoking habit (63 vs. 57%; *P* = 0.01), renal impairment (16 vs. 11%; *P* = 0.009), and a higher GRACE score (132 vs. 91; *P* < 0.001) compared with those with a normal cardiac troponin concentration. In addition, among those who were randomized to the early CTCA group and underwent CTCA, patients with an elevated cardiac troponin concentration were more likely to have obstructive coronary artery disease (59 vs. 33%; *P* < 0.001).

**Table 1:** Baseline demographics and clinical characteristics by presentation cardiac troponin

	Elevated cardiac troponin	Normal cardiac troponin	*P-*value
	(*N* = 1004)	(*N* = 744)	
Age (years)	61 (52–72)	61 (53–70)	0.31
Sex, male	674 (67)	440 (59)	<0.001
Diabetes mellitus	193 (19)	125 (17)	0.19
Hypertension	455 (45)	362 (49)	0.17
Dyslipidaemia	375 (37)	319 (43)	0.02
Former or current smoker	635 (63)	426 (57)	0.01
Family history of premature coronary artery disease	287 (29)	252 (34)	0.02
Estimated glomerular filtration rate 30–59 mL/min/1.73 m^2^	156 (16)	83 (11)	0.009
Prior coronary artery disease	250 (25)	351 (47)	<0.001
Abnormal electrocardiogram	607 (60)	457 (61)	0.68
GRACE score	132 (109–151)	91 (73–109)	<0.001
Killip, class I	979 (98)	726 (98)	0.90
Randomized to the early CTCA group	492 (49)	385 (52)	0.26
CTCA finding^a^			<0.001
Normal coronary arteries	69 (16)	109 (32)	
Non-obstructive coronary artery disease	104 (25)	118 (35)	
Obstructive coronary artery disease	249 (59)	110 (33)	
Coronary calcium score^a^	119 (6–602)	32 (0–247)	<0.001

CTCA, computed tomography coronary angiography; GRACE, Global Registry of Acute Coronary Events.

a Data are limited to patients in the early CTCA group who have available data.

### Non-invasive cardiovascular testing

**Figure 1:**
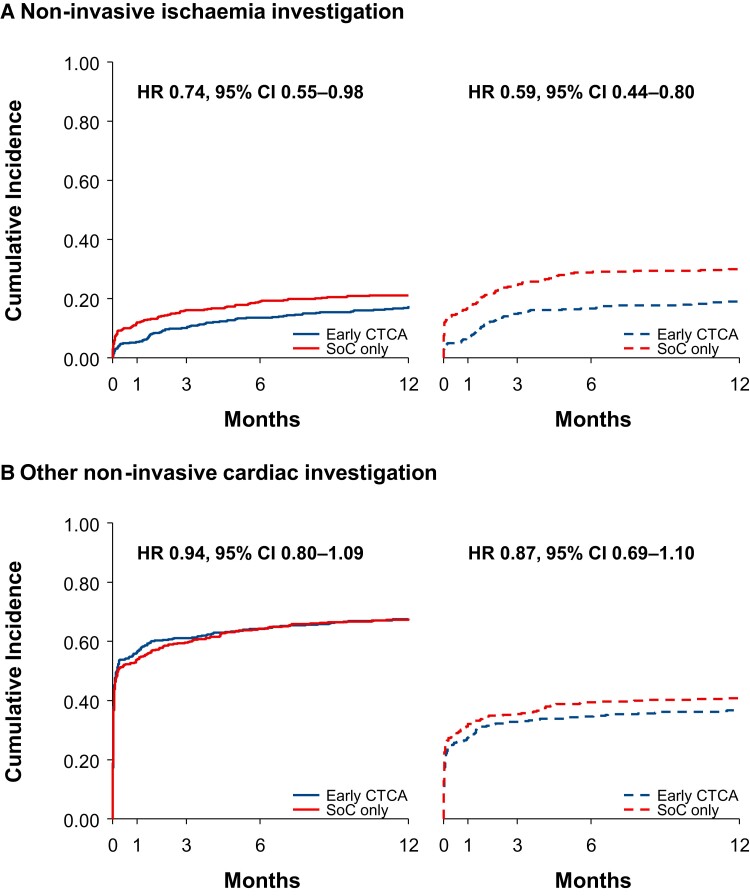
Cumulative incidence for non-invasive cardiovascular testing. Kaplan–Meier estimates for non-invasive ischaemia (*A*) and other non-invasive cardiac (*B*) investigations stratified by the presence (solid lines) or absence (dashed lines) of cardiac troponin elevation at presentation and by trial assignment. CI, confidence interval; HR, hazard ratio.

During follow-up, non-invasive cardiovascular testing was performed in 727 (72%) patients with an elevated cardiac troponin concentration and 384 (52%) with a normal concentration (hazard ratio 1.53, 95% confidence interval 1.29–1.82; *P* < 0.001; see Supplementary material online, *Figure S1A*). The rate of non-invasive cardiovascular testing (see Supplementary material online, *Table S2*) was consistently lower in the early CTCA group among those with an elevated (hazard ratio 0.88, 95% confidence interval 0.76–1.02) or a normal (hazard ratio 0.78, 95% confidence interval 0.64–0.95; *P*_interaction_ = 0.33) cardiac troponin concentration (see Supplementary material online, *Figure S1B* and *C*). The rate of non-invasive ischaemia investigations was similar between those with an elevated cardiac troponin concentration and a normal concentration (hazard ratio 0.89, 95% confidence interval 0.67–1.17; *P* = 0.39; see Supplementary material online, *Figure S2A*) but was again consistently reduced by early CTCA in those with an elevated cardiac troponin concentration (hazard ratio 0.74, 95% confidence interval 0.55–0.98) and a normal (hazard ratio 0.59, 95% confidence interval 0.44–0.80) concentration (*Figure 1A*; *P*_interaction_ = 0.31). Although other non-invasive cardiac investigations were more frequently performed in those with an elevated rather than a normal cardiac troponin concentration (hazard ratio 1.87, 95% confidence interval 1.55–2.26; *P* < 0.001; see Supplementary material online, *Figure S2B*), the rate of other non-invasive cardiac investigations was comparable between trial assignment in those with an elevated cardiac troponin concentration and a normal concentration (*Figure 1B*; *P*_interaction_ = 0.62).

### Invasive coronary angiography and coronary revascularization

**Figure 2:**
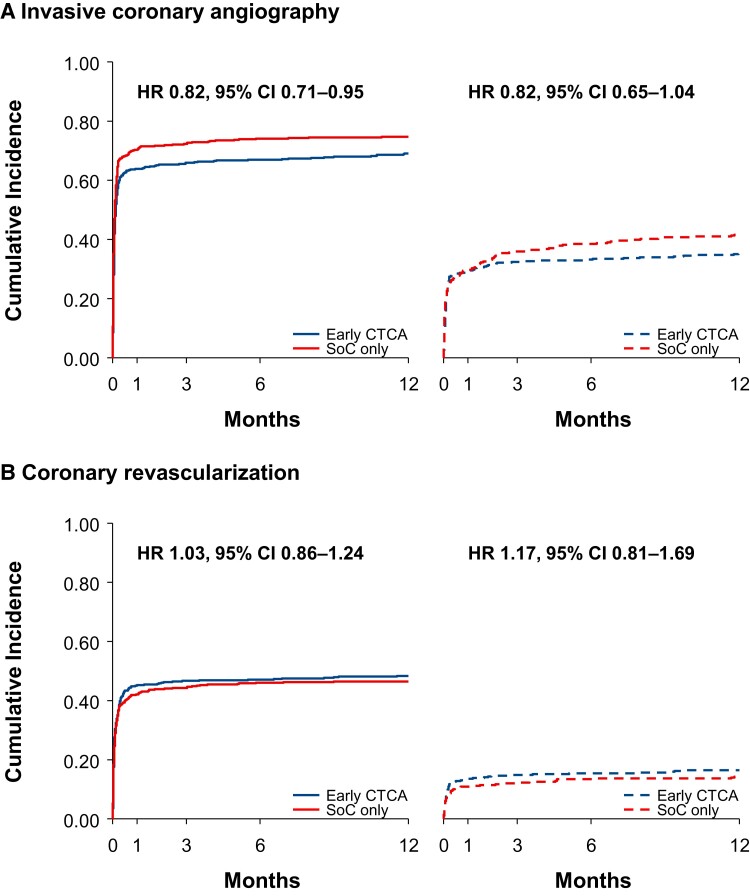
Cumulative incidence for invasive coronary management. Kaplan–Meier estimates for invasive coronary angiography (*A*) and coronary revascularization (*B*) stratified by the presence (solid lines) or absence (dashed lines) of cardiac troponin elevation at presentation and by trial assignment. CI, confidence interval; HR, hazard ratio.

Patients with an elevated cardiac troponin concentration had double the rate of invasive coronary angiography (72 vs. 38%; hazard ratio 2.56, 95% confidence interval 2.13–3.06; *P* < 0.001) and approximately three times the rate of coronary revascularization (47 vs. 15%; hazard ratio 3.15, 95% confidence interval 2.44–4.07; *P* < 0.001) compared with patients with a normal cardiac troponin concentration (see Supplementary material online, *Figure S3*). The rate of invasive coronary angiography was consistently lower in the early CTCA group than in the standard of care only group among those with (hazard ratio 0.82, 95% confidence interval 0.71–0.95) and without (hazard ratio 0.82, 95% confidence interval 0.65–1.04) cardiac troponin elevation (*Figure 2A*; *P*_interaction_ = 0.99). The reduction of invasive coronary angiography by early CTCA was comparable across the first 30 days but proportionately appeared to be more marked beyond 30 days in those with a normal cardiac troponin concentration (*Table 2*). Although there was no heterogeneity in the rates of coronary revascularization between trial assignment by cardiac troponin concentration (*Figure 2B*; *P*_interaction_ = 0.57), coronary revascularization was more frequently performed in the early CTCA group than in the standard of care only group among those referred to invasive coronary angiography (*Table 3*).

**Table 2:** Landmark analysis on rates of invasive coronary angiography by presentation cardiac troponin

	Follow-up ≤30 days				Follow-up >30 days			
	Early CTCA	SoC only	Hazard ratio (95% confidence interval)	*P*-value^a^	Early CTCA	SoC only	Hazard ratio (95% confidence interval)	*P-*value^a^
Elevated cardiac troponin	313/492 (64)	359/512 (70)	0.82	0.38	25/176 (14)	22/151 (15)	0.91	0.14
			(0.70–0.95)				(0.51–1.63)	
Normal cardiac troponin	111/384 (29)	106/358 (30)	0.94		24/272 (9)	42/250 (17)	0.51	
			(0.72–1.23)				(0.31–0.85)	

CTCA, computed tomography coronary angiography; SoC, standard of care.

a Interactions between trial assignment and presentation cardiac troponin were tested.

**Table 3:** Rates of coronary revascularization among patients referred to invasive coronary angiography by presentation cardiac troponin

	Elevated cardiac troponin			Normal cardiac troponin			*P*-value^a^
	Early CTCA (*N* = 338)	SoC only (*N* = 381)	Odds ratio (95% confidence interval)	Early CTCA (*N* = 136)	SoC only (*N* = 149)	Odds ratio (95% confidence interval)	
PCI	201 (59)	197 (52)	1.43 (1.05–1.93)	59 (43)	43 (29)	1.91 (1.16–3.15)	0.33
CABG surgery	44 (13)	46 (12)	1.01 (0.64–1.60)	8 (6)	9 (6)	1.08 (0.40–2.93)	0.90
PCI or CABG surgery	236 (70)	237 (62)	1.43 (1.04–1.98)	64 (47)	51 (34)	1.83 (1.12–3.00)	0.42

CABG, coronary artery bypass graft; CTCA, computed tomography coronary angiography; PCI, percutaneous coronary intervention; SoC, standard of care.

a Interactions between trial assignment and presentation cardiac troponin were tested.

### Primary outcome

**Figure 3:**
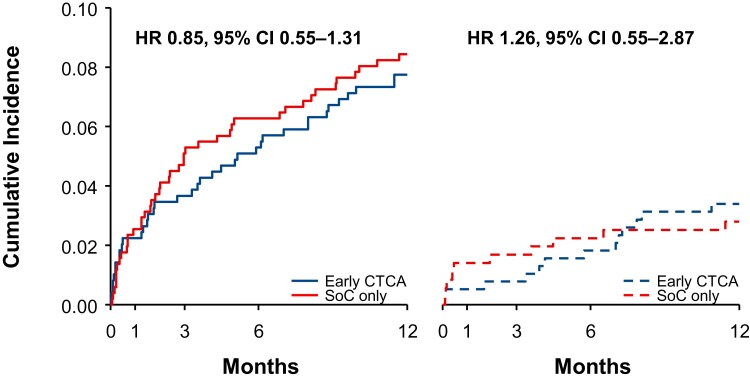
Cumulative incidence for death or subsequent Type 1 or 4b myocardial infarction. Kaplan–Meier estimates stratified by the presence (solid lines) or absence (dashed lines) of cardiac troponin elevation at presentation and by trial assignment. CTCA, computed tomography coronary angiography; SoC, standard of care.

At 1 year, the rate of death or subsequent Type 1 or 4b myocardial infarction was substantially higher among patients with an elevated cardiac troponin concentration (8 vs. 3%; hazard ratio 2.31, 95% confidence interval 1.25–4.25; *P* = 0.007; see Supplementary material online, *Figure S4*). For the relative effect of early CTCA on the primary outcome, there was no interaction (*P*_interaction_ = 0.41) between those with an elevated cardiac troponin concentration (hazard ratio 0.85, 95% confidence interval 0.55–1.31) and a normal (hazard ratio 1.26; 95% confidence interval 0.55–2.87) concentration (*Figure 3*).

### Sensitivity analyses

The restriction of our analysis to the 28 sites using a high-sensitivity cardiac troponin assay since trial inception demonstrated findings that were consistent with the main analysis with no major demonstrable differences in outcomes (see Supplementary material online, *Table S3*). In contrast, early CTCA appeared to have a heterogenous impact on non-invasive ischaemia investigations. Compared with those who had a discharge diagnosis of myocardial infarction, there was a halving in the rate of non-invasive ischaemia investigations in patients with an elevated cardiac troponin concentration who had a discharge diagnosis other than myocardial infarction (*P*_interaction_ = 0.003; see Supplementary material online, *Table S4*).

## Discussion

In this pre-specified secondary analysis of the RAPID-CTCA trial, patients with suspected acute coronary syndrome and an elevated cardiac troponin concentration at presentation had higher rates of non-invasive and invasive testing, coronary revascularization, and death or subsequent Type 1 or 4b myocardial infarction at 1 year. Early CTCA reduced the use of non-invasive cardiovascular testing and non-invasive ischaemia investigations, and invasive coronary angiography without altering the use of coronary revascularization and the primary outcome regardless of cardiac troponin elevation. This reconfirms that cardiac troponin is an excellent diagnostic and prognostic biomarker that is associated with the greater use of investigations and treatments for patients with acute coronary syndrome. However, reductions in the rates of non-invasive ischaemia investigations and invasive coronary angiography associated with CTCA were seen irrespective of cardiac troponin concentration, suggesting that early CTCA safely reduces downstream investigations regardless of the presence of myocardial injury or infarction.

Guidelines have suggested the use of CTCA in those at low risk of coronary artery disease. It would, therefore, be expected that early CTCA would have been particularly useful in patients with acute chest pain and no cardiac troponin elevation. In our population of patients with a normal cardiac troponin concentration at presentation, the median GRACE score was 91, and the primary event rate was 3% at 1 year, thereby confirming their lower risk status. Indeed, two-thirds of patients had either normal coronary arteries or non-obstructive coronary artery disease, consistent with our recent observation.^16^ We would, therefore, have anticipated greater benefit of CTCA in those with a normal cardiac troponin concentration. However, we identified no evidence of heterogeneity in any trial outcomes by the presence or absence of cardiac troponin elevation at presentation.

Earlier studies focusing on patients with low-risk acute chest pain who had a normal cardiac troponin concentration suggested that incorporating CTCA into the initial management pathway in emergency departments yielded a different clinical trajectory,^17^ being associated with a more rapid diagnosis, shorter lengths of stay, and higher rates of invasive coronary angiography and coronary revascularization.^18–20^ However, the introduction of more sensitive cardiac troponin assays into contemporary clinical pathways for acute coronary syndrome has challenged the role of CTCA in the initial management of such patients. Indeed, in the Better Evaluation of Acute Chest Pain with Coronary Computed Tomography Angiography (BEACON) trial, early CTCA did not result in differences in length of stay or rates of hospitalization, invasive coronary angiography, and coronary revascularization among patients with a cardiac troponin concentration ≤3 times the 99th centile upper reference limit.^21^ This partly reflects the low-positive predictive value of modest cardiac troponin elevations for obstructive coronary artery disease and Type 1 myocardial infarction.^22,23^ In our analysis, patients with a normal cardiac troponin concentration at presentation were less likely to undergo further non-invasive ischaemia investigations or invasive coronary angiography when assessed by CTCA. This was most prominent beyond 30 days where rates of invasive angiography were halved by early CTCA. This reduction rather than an increase in the rates of invasive coronary angiography may also reflect differences in risk of the populations studied and duration of follow-up.^15^

We observed that patients who were randomized to early CTCA and subsequently underwent invasive coronary angiography were proportionately more likely to undergo coronary revascularization. This was particularly true for patients without cardiac troponin elevation with a two-fold increase in the likelihood of percutaneous coronary intervention. Given that there were no differences in the overall rates of coronary revascularization by trial assignment, this reflects the identification of normal or minimally diseased coronary arteries by CTCA such that only those with potentially obstructive coronary artery disease underwent invasive coronary angiography. This, therefore, enables a more efficient use of cardiac catheterization facilities by accurately selecting candidates who may benefit from coronary revascularization.

For patients with acute coronary syndrome and an elevated cardiac troponin concentration, current guidelines favour invasive coronary angiography as the primary tool to visualize coronary artery anatomy and to decide the optimal revascularization strategy. Indeed, one guideline recommends this should be performed within 24 h of presentation.^24^ However, CTCA is diagnostically consistent with, and prognostically equivalent to, invasive coronary angiography in patients with non-ST-segment elevation acute coronary syndrome.^25,26^ In the role of initial CARdiovascular Magnetic rEsoNance imaging and computed Tomography Angiography in non-ST-elevation myocardial infarction patients (CARMENTA) trial, a CTCA-first strategy reduced the immediate and overall referral for invasive coronary angiography by a third without an increase in the rate of major cardiac or procedure-related adverse events at 1 year.^27^ Consistent with this, our analysis showed an 18% reduction in the likelihood of invasive coronary angiography with similar rates of coronary revascularization irrespective of cardiac troponin concentration. Again, in those referred for invasive coronary angiography, coronary revascularization was more likely in the early CTCA group, indicating that early CTCA avoids unnecessary invasive coronary angiography and allows for efficient identification of those who might benefit from coronary revascularization, even in those with an elevated cardiac troponin concentration. Our findings, along with those from the CARMENTA trial, suggest that CTCA does assist in the selection of patients for invasive coronary angiography and coronary revascularization in patients with an elevated cardiac troponin concentration. This contrasts with the 2020 European Society of Cardiology guidelines, which recommend the use of CTCA only in those with normal or inconclusive cardiac troponin concentrations.^24^

The use of high-sensitivity cardiac troponin assays has increased the numbers of patients with acute chest pain who have an elevated cardiac troponin concentration. Many of these patients have small elevations and do not have myocardial infarction but acute or chronic myocardial injury. It can be very challenging to identify which patients have myocardial infarction and which have myocardial injury. Indeed, many patients undergoing invasive coronary angiography with a provisional diagnosis of myocardial infarction have no obstructive coronary artery disease.^6,28^ In our analysis, we found that a third of patients with an elevated cardiac troponin concentration at presentation were ultimately discharged without a diagnosis of myocardial infarction. In this group of patients, early CTCA appeared to be particularly useful and halved the rates of non-invasive ischaemia testing and invasive coronary angiography. Thus, where there is uncertainty regarding the diagnosis of myocardial infarction in a patient with cardiac troponin elevation, early CTCA appears to be particularly helpful in avoiding further non-invasive and invasive testing.

In the CArdiac cT in the treatment of acute CHest pain (CATCH) trial, early CTCA improved clinical outcomes at a median of 18 months in those with acute chest pain and a normal cardiac troponin concentration.^29^ Although the extent and burden of coronary artery disease predicts adverse outcomes in patients with acute coronary syndrome independently of cardiac biomarkers,^30^ we found no differences in the rate of death or subsequent Type 1 or 4b myocardial infarction at 1 year either across the whole trial population or within these subgroups stratified by cardiac troponin elevation. Whether differences may emerge with longer-term follow-up remains to be seen.

Our study has a number of limitations which we should highlight. First, although this study is a pre-specified secondary analysis, randomization was not stratified by cardiac troponin, and we evaluated only the presentation concentration rather than the complete profile of serial of cardiac troponin measurements. Cardiac troponin assays employed in the RAPID-CTCA trial varied by site, and not all sites used a high-sensitivity assay or the 99th centile upper reference limit recommended by the guidelines. However, most sites used both, and our sensitivity analysis that was restricted to those sites where high-sensitivity cardiac troponin testing was performed throughout the trial demonstrated the consistency of our findings. In addition, cardiac troponin concentrations below the diagnostic threshold for myocardial infarction provide further prognostic information, and whether this can be used in conjunction with CTCA to improve long-term outcomes is unknown. This is currently being investigated in the Troponin in Acute chest pain to Risk stratify and Guide EffecTive use of Computed Tomography Coronary Angiography (TARGET-CTCA) trial (ClinicalTrials.gov identifier, NCT03952351). Furthermore, the RAPID-CTCA trial entry criteria required enrichment for risk, and patients were required to have an elevated cardiac troponin concentration, an abnormal electrocardiogram, or a prior history of coronary artery disease. As such, our findings were restricted to this population and might not be representative of the broader population of patients with acute chest pain who present to the Emergency Department. We should also acknowledge that the RAPID-CTCA trial was powered to assess the effect of early CTCA on the primary outcome. Given that there were no differences in the rate of the primary outcome, our findings on secondary and *post hoc* outcomes were hypothesis-generating in nature. Finally, as with any open trial, there is a likelihood for bias in the RAPID-CTCA trial.

## Conclusion

Presentation cardiac troponin does not influence the impact of early CTCA on rates of non-invasive testing, invasive coronary angiography, coronary revascularization, or clinical outcomes in intermediate-risk patients with suspected acute coronary syndrome. Our findings suggest that decisions regarding the early use of CTCA in these patients should not be based upon cardiac troponin alone.

## Supplementary Material

zuac057_Supplementary_Data

## Data Availability

De-identified data are available on reasonable request which should be submitted to the Chief Investigator of the RAPID CTCA trial—Prof Alasdair J Gray.
