# Supplementary material for: Presentation cardiac troponin and early computed tomography coronary angiography in patients with suspected acute coronary syndrome: a pre-specified secondary analysis of the RAPID-CTCA trial
Source: Eur Heart J Acute Cardiovasc Care. 2022 Jun 1;11(7):570–9. doi: 10.1093/ehjacc/zuac057 (PMC9302931; doi:10.1093/ehjacc/zuac057)

**Supplementary File**

**Presentation Cardiac Troponin and Early Computed Tomography Coronary Angiography in Patients with Suspected Acute Coronary Syndrome**

**A pre-specified secondary analysis of the RAPID-CTCA trial**

Kang-Ling Wang, Carl Roobottom, Jason E Smith, Steve Goodacre, Katherine Oatey, Rachel O’Brien, Robert F Storey, Nick Curzen, Liza Keating, Attila Kardos, Dirk Felmeden, Praveen Thokala, Nicholas L Mills, David E Newby, and Alasdair J Gray, on behalf of the RAPID-CTCA Investigators

**Table of Contents**

**Appendix 1 – Committees, Members, and Investigators**

**Appendix 2 – Supplementary Tables**

Supplementary Table 1. Cardiac troponin assays and reference values

Supplementary Table 2. Rates of study outcomes among patients by presentation cardiac troponin concentration and by trial assignment

Supplementary Table 3. Sensitivity analysis on data restricted to sites using a high-sensitivity cardiac troponin assay since trial inception

Supplementary Table 4. Sensitivity analysis on data restricted to patients who had cardiac troponin elevation at presentation by discharge diagnosis

**Appendix 3 – Supplementary Figures**

Supplementary Figure 1. Cumulative incidence for overall non-invasive cardiovascular testing

Supplementary Figure 2. Cumulative incidence for non-invasive cardiovascular testing by category and by cardiac troponin concentration at presentation

Supplementary Figure 3. Cumulative incidence for invasive coronary management by cardiac troponin concentration at presentation

Supplementary Figure 4. Cumulative incidence for death or subsequent type 1 or 4b myocardial infarction by cardiac troponin concentration at presentation

**Steering Committee**

Prof Tim Coats (Chairperson, University of Leicester), Prof Alasdair Gray (Chief Investigator, University of Edinburgh), Prof Steff Lewis (Trial Statistician, University of Edinburgh), Mr Kenneth Archibald (Layperson), Mr Graham Bell (Layperson), Mr Rodney Mycock (Layperson), Dr Russell Bull (Royal Bournemouth and Christchurch Hospitals NHS Foundation Trust), Prof Gerry McCann (University of Leicester), Dr Tarun Mittal (Royal Brompton and Harefield NHS Foundation Trust), and Dr James Rudd (University of Cambridge).

**Data Monitoring Committee**

Prof Carrol Gamble (Chairperson, University of Liverpool), Prof Simon Carley (Manchester University NHS Foundation Trust), and Prof Simon Padley (Royal Brompton and Harefield NHS Foundation Trust).

**Endpoint Adjudication Committee**

Prof Nicholas Mills (University of Edinburgh), Dr Anoop Shah (London School of Hygiene and Tropical Medicine), and Dr Andrew Chapman (University of Edinburgh).

**CTCA Quality Assurance Group**

University Hospitals Plymouth NHS Trust – Prof Carl Roobottom, Dr Gareth Morgan-Hughes, Dr Vikram Raju, and Ms Kym Luke.

Royal Infirmary of Edinburgh – Dr Mark Jones and Dr Fergus Perks.

**Participating Centres, Principal Investigators, and Research Teams**

**Edinburgh – Royal Infirmary of Edinburgh** – Prof Alasdair Gray – Polly Black, Caroline Blackstock, Julia Grahamslaw, Mark Jones, Collette Keanie, Margaret MacLeod, Siobhan McLaughlin, Rachel O’Brien, Fergus Perks, Alyson Phillips, Ewan Pirie, Janet Summerside, Gordon Truong, Kirsty Weston, and Jennifer Wooton.

**Sheffield – Northern General Hospital** – Prof Steve Goodacre – Sarah Bird, Peter Brown, Hridesh Chatha, Alan Fletcher, Catherine Hill, Shery Mofidi, Hasan Qayyum, Robert Storey, Judith Sugden, and Anna Wilson.

**Plymouth – Derriford Hospital** – Prof Carl Roobottom and Prof Jason Smith – Alison Jeffrey, Memory Mwadeyi, Rosalyn Squire, and Peter Wafer.

**Torquay – Torbay Hospital** – Dr Dirk Feldman – Lesley Archer, Lisa Felmeden, Guy Gribbin, Sarah Harrison, Debbie Hughes, Philip Keeling, Ian Mahy, Allison Summerhayes, Justine Sutton, and Abdullah Yonis.

**Kirkcaldy – Victoria Hospital** – Dr Andrew Kinnon and Dr Ajay Yerramasu – Susan Fowler, Amanda McGregor, Karen Grey, Tom Hartley, David Szapiro, Lorraine Dinnel, and Dennis Sandeman.

**Dudley – Russells Hall Hospital** – Dr Robert Huggett – Julie Dean and Amy Pugh.

**Reading – Royal Berkshire Hospital** – Dr Liza Keating – Parminder Bhuie, James Briggs, Claire Burnett, Abby Gandy, Nicola Jacques, Sarah MacGill, Archie Speirs, and Niamh Tolan.

**Bradford – Bradford Royal Infirmary** – Dr Sudantha Bulugahapitiya – Craig Atkinson, Mark Kon, Carita Krannila, and Manitha Thomas.

**Bournemouth – Royal Bournemouth Hospital** – Dr Jehangir Din – Russell Bull, Stephanie Horler, Nicki Lakeman, Jane McLeod, Sara Nix, and Sue Thomas.

**St Helier – Jersey General Hospital** – Dr Andrew Mitchell – Daniel Ahlert, Christopher Edmond, Christopher Hare, Kelly Anne Kinsella, Jessica Langtree, James Speakman, and Ranjit Thomas.

**Melrose – Borders General Hospital** – Dr Anne Scott – Gillian Donaldson, Fiona Hall, Terry Fairbairn, and Christopher Rofe.

**Newcastle – Royal Victoria Infirmary** – Dr Anna Beattie – Jennifer Adams-Hall, Ange Bailey, Kris Bailey, Leslie Bremner, Ifti Haq, and Angela Phillipson.

**London – University Hospital Lewisham** – Dr Khalid Alfakih – Saroj David, Osman Najam, and Samia Pilgrim.

**Glasgow – Glasgow Royal Infirmary** – Dr Adrian Brady – Claire Adams, Ammani Brown, Andrew Dougherty, Ailsa Geddes, Karen Lang, David Lowe, Ross MacDuff, Lorraine McGregor, Giles Roditi, Susan Thornton, and Joyce Triscott.

**Milton Keynes – Milton Keynes University Hospital** – Prof Attila Kardos – Felicia Adjei, Antoanela Colda, Caitlin Chapman, Veronica Edgell, Michael Fell, Laszlo Halmai, Aarzoo Khan, John Northfield, Cheryl Padilla-Harris, Mike Pashler, Gill Richie, Diane Scaletta, Sarah–Beth Sunderland, Joanne Turner, Lois Vickery, Sonya Walia, Felicity Williams, Lynn Wren, and Nicola Wright.

**Stoke-on-Trent – Royal Stoke University Hospital** – Dr Hefin Jones – Holly Maguire and Resti Varquez.

**West Bromwich – Sandwell General Hospital** – Dr Derek Connolly – Anthony D’Sa, Vinoda Sharma, and Ashley Turner.

**London – St Thomas' Hospital** – Dr Ronak Rajani – Megan Bell, Giulia Benedetti, Kirsty Gibson, Sze Mun Mak, Rebecca Preston, Amy Raynsford, and Ruth Sanchez-Vidal.

**Rotherham – Rotherham Hospital** – Dr Rangasamy Muthusamy and Dr Simon Smith – Susan Biggins, Kathryn Dixon, Peter Kraut, Mwada Lawan, Victoria Murray, Tom Mwambingu, Rachel Walker, and Carol Weston.

**Leeds – Leeds General Infirmary** – Dr Abdel-Rahman Saif-El-Dean – Roo Byrom-Goulthorp, Michael Darby, Eunice Ikongo, Annette Johnstone, Alan Lin, and Melanie Mcginlay.

**Birmingham – Queen Elizabeth Hospital Birmingham** – Dr Ben Holloway – Tania Albutt, Vicky Dawson, Claire Dowling, Karen Isaacs, Cheyanne Kaila, Gareth Lewis, Nicky Mortimer, Sunitha San, Kelly Tabor, and Kealy Wright.

**Redhill – East Surrey Hospital** – Dr Ansuman Saha – Riaz Ahmed, Sally Collins, Sarah Davies, and Nokukhanya Ndlovu.

**Southampton – Southampton General Hospital** – Dr Nick Curzen – Ausami Abbas, Alison Calver, Simon Corbett, Peter Cowburn, Andrew Flett, Huon Gray, Stephen Harden, Paul Haydock, Michael Mahmoudi, John Paisey, Charles Peebles, Drew Rakhit, John Rawlins, Paul Roberts, Benoy Shah, James Shambrook, Iain Simpson, Rohit Sirohi, Wagas Ullah, Katharine Vedwan, James Wilkinson, and Arthur Yue.

**Manchester – Wythenshawe Hospital** – Dr Matthias Schmitt – Sarra Giannopoulou, Melanie Greaves, Stephen McGlynn, Chris Miller, Lindsay Murray, Akhila Muthuswamy, and Anie Nicholas.

**Luton – Luton and Dunstable University Hospital** – Dr Christopher Travill – Susan Gent and Nafisa Hussain.

**London – Royal London Hospital and Whipps Cross University Hospital** – Dr Ceri Davies, Prof Tim Harris, and Dr Ben Bloom – Raine Astin-Chamberlain, Olivia Bolton, Dan Martin, Lyrics Noba, Georgia Norman, Shelley Page, Helen Power, Imogen Skene, David Smith, and Jon Walters.

**Worcester – Worcestershire Royal Hospital** – Dr Will Roberts – Angela Doughty, Elaine Byng-Hollander, and Helen Routledge.

**Belfast – Ulster Hospital** – Dr Patrick Donnelly – Leah Hammond, Jayne Hutchinson, Stephanie Kelly, Susan Regan, and Aileen Smith.

**Stockton-on-Tees – University Hospital of North Tees** – Dr Justin Carter – Julie Gray, Sarah Purvis, and Pam Race.

**Dundee – Ninewells Hospital** – Dr John Irving – Christine Almaden-Boyle, Kim Bissett, Carol Blues, Jackie Duff, Scot Dundas, Shirley Fawcett, Graeme Houston, Emma Hutchison, Debbie Letham, Ann Mackintosh, Laura Meach, Laura Jayne Queripcz, and Alan Webster.

**Portsmouth – Queen Alexandra Hospital** – Dr Chris Vorwerk – Julian Atchley, Zoe Daly, and Kat Ellinor.

**Wrexham – Wrexham Maelor Hospital** – Dr Ash Basu – Richard Cowell, Helen Craddock, Rachel Hughes, Lynda Sackett, Victoria Saul, Fiona Smith, Jane Stockport, and Clare Watkins.

**Basildon – Basildon University Hospital** – Dr Jason Dungu – Edward Barden, Jackie Colnet, Swamy Gedeza, Laura Hoskin, Lauren Kittridge, Gracie Maloney, Claire McCormick, Anne Nicholson, Stacey Pepper, Joanne Riches, and Annaliza Sevillano.

**Wolverhampton – New Cross Hospital** – Dr Elisa McAlindon, Dr Sandeep Hothi, Dr David Rosewarne, and Dr Arivalagan Bapusamy – Vincent Amoah, Stacey Aulton, Victoria Cottam, Stella Metherell, Sarah Milgate, Elizabeth Radford, and Andy Smallwood.

**Inverness – Raigmore Hospital** – Dr Jonathan Watt – Charlotte Barr, Jonathan Broadie, David Eason, Ing-Marie Logie, Debbie McDonald, Laura O’Keeffe, Donna Patience, and Lesley Patience.

**Glasgow – Queen Elizabeth University Hospital** – Dr Claire McGroarty – Faheem Ahmad, Nicola Baxter, Ammani Brown, John Byrne, Damien Collison, Tracey Hopkins, Hayley King, David Lowe, Evonne McLennan, Giles Roditi, David Stobo, Mark Wilson, and Rosie Woodward.

| **Supplementary Table 1. Cardiac troponin assays and reference values.** | | |
| --- | --- | --- |
|  | **Type of cardiac troponin assay** | **99^th^ centile upper reference limit** |
| Royal Infirmary of Edinburgh, Edinburgh | High-sensitivity/I | 34 (M)  16 (F) |
| Northern General Hospital, Sheffield | High-sensitivity/T | 14 |
| Derriford Hospital, Plymouth | High-sensitivity/T | 13 |
| Torbay Hospital, Torquay | High-sensitivity/T | 14 |
| Victoria Hospital, Kirkcaldy | High-sensitivity/T | 13 |
| Russells Hall Hospital, Dudley | High-sensitivity/T | 15 (M)  10 (F) |
| Royal Berkshire Hospital, Reading | High-sensitivity/T | 14 |
| Bradford Royal Infirmary, Bradford | High-sensitivity/I | 50 |
| Royal Bournemouth Hospital, Bournemouth | High-sensitivity/T | 14 |
| Jersey General Hospital, St Helier | High-sensitivity/I | 33 |
| Borders General Hospital, Melrose | Contemporary/I | 50 |
| Royal Victoria Infirmary, Newcastle | High-sensitivity/T | 14 (M)  9 (F) |
| University Hospital Lewisham, London | Contemporary/I | 100 |
| Glasgow Royal Infirmary, Glasgow | High-sensitivity/I | 33 |
| Milton Keynes University Hospital, Milton Keynes | Contemporary/I | 10 |
| Royal Stoke University Hospital, Stoke-on-Trent | High-sensitivity/I | 39 |
| Sandwell General Hospital, West Bromwich | High-sensitivity/T | 14 |
| St Thomas' Hospital, London | High-sensitivity/T | 13 |
| Rotherham Hospital, Rotherham | Contemporary/I | 39 |
| Leeds General Infirmary, Leeds | High-sensitivity/I | 49 |
| Queen Elizabeth Hospital Birmingham, Birmingham | High-sensitivity/T | 14 |
| East Surrey Hospital, Redhill | High-sensitivity/T | 14 |
| Southampton General Hospital, Southampton | High-sensitivity/I | 39 |
| Wythenshawe Hospital, Manchester | High-sensitivity/I | 33 (M)  15 (F) |
| Luton and Dunstable University Hospital, Luton | High-sensitivity/T | 14 |
| Royal London Hospital, London  Laboratory assay  Point of care assay | High-sensitivity/T  Contemporary/I | 14  23 |
| Whipps Cross University Hospital, London | Contemporary/I | 39 |
| Worcestershire Royal Hospital, Worcester | High-sensitivity/T | 20 |
| Ulster Hospital, Belfast | High-sensitivity/T | 14 |
| University Hospital of North Tees, Stockton-on-Tees | High-sensitivity/I | 39 |
| Ninewells Hospital, Dundee | Contemporary/I | 44 |
| Queen Alexandra Hospital, Portsmouth | High-sensitivity/I | 39 |
| Wrexham Maelor Hospital, Wrexham | Contemporary/I | 40 |
| Basildon University Hospital, Basildon | High-sensitivity/T | 14 |
| New Cross Hospital, Wolverhampton | High-sensitivity/I | 34 (M)  15 (F) |
| Raigmore Hospital, Inverness | Contemporary/I | 40 |
| Queen Elizabeth University Hospital, Glasgow | High-sensitivity/I | 33 (M)  15 (F) |
| * All upper reference limits were in ng/L, and whether to use a uniform or sex-specific thresholds was determined by local laboratory reference standards and clinical care pathways.  † Cardiac troponin assays changed to high-sensitivity cardiac troponin I (in Derriford Hospital and Raigmore Hospital) and to high-sensitivity cardiac troponin T (in University Hospital Lewisham) assays during the trial. | | |

| **Supplementary Table 2. Rates of study outcomes among patients by presentation cardiac troponin concentration and by trial assignment.** | | | | |
| --- | --- | --- | --- | --- |
|  | **Elevated cardiac troponin** | | **Normal cardiac troponin** | |
|  | Early CTCA  (N = 492) | SoC only  (N = 512) | Early CTCA  (N = 385) | SoC only  (N = 359) |
| Non-invasive cardiovascular testing | 352 (72) | 375 (73) | 185 (48) | 199 (55) |
| Non-invasive ischaemia investigation | 85 (17) | 107 (21) | 73 (19) | 108 (30) |
| Other non-invasive cardiac investigation | 331 (67) | 345 (67) | 141 (37) | 146 (41) |
| Invasive coronary angiography | 338 (69) | 381 (74) | 136 (35) | 149 (42) |
| Coronary revascularisation | 236 (48) | 237 (46) | 64 (17) | 51 (14) |
| Death or subsequent type 1 or 4b myocardial infarction | 38 (8) | 43 (8) | 13 (3) | 10 (3) |
| * Data were n (%).  CTCA = computed tomography coronary angiography; SoC = standard of care | | | | |

| **Supplementary Table 3. Sensitivity analysis on data restricted to sites using a high-sensitivity cardiac troponin assay since trial inception.** | | | |
| --- | --- | --- | --- |
|  | **Hazard Ratio (95% Confidence Interval)** | | **p value‡** |
|  | **Elevated cardiac troponin**  **(N = 817)** | **Normal cardiac troponin**  **(N = 629)** |  |
| Non-invasive cardiovascular testing | 0.87 (0.74 to 1.03) | 0.68 (0.55 to 0.85) | 0.09 |
| Non-invasive ischaemia investigation | 0.71 (0.51 to 1.00) | 0.48 (0.34 to 0.68) | 0.11 |
| Other non-invasive cardiac investigation | 0.94 (0.79 to 1.11) | 0.81 (0.63 to 1.05) | 0.37 |
| Invasive coronary angiography | 0.81 (0.69 to 0.95) | 0.77 (0.60 to 0.98) | 0.73 |
| Coronary revascularisation | 0.96 (0.79 to 1.18) | 1.11 (0.75 to 1.65) | 0.53 |
| Death or subsequent type 1 or 4b myocardial infarction | 0.73 (0.45 to 1.20) | 1.32 (0.53 to 3.30) | 0.26 |
| * Effect estimates compared between the early computed tomography coronary angiography group and the standard of care only group.  † Models were adjusted for study site (as a random effect), Global Registry of Acute Coronary Events score (using a restricted cubic spline), and prior coronary artery disease.  ‡ Interactions between trial assignment and presentation cardiac troponin were tested. | | | |

| **Supplementary Table 4. Sensitivity analysis on data restricted to patients who had cardiac troponin elevation at presentation by discharge diagnosis.** | | | |
| --- | --- | --- | --- |
|  | **Odds Ratio (95% Confidence Interval)** | | **p value‡** |
|  | **Myocardial infarction**  **(N = 665)** | **Others**  **(N = 339)** |  |
| Non-invasive cardiovascular testing | 0.99 (0.66 to 1.47) | 0.50 (0.30 to 0.84) | 0.04 |
| Non-invasive ischaemia investigation | 1.19 (0.77 to 1.85) | 0.41 (0.24 to 0.71) | 0.003 |
| Other non-invasive cardiac investigation | 0.93 (0.64 to 1.37) | 0.71 (0.43 to 1.15) | 0.38 |
| Invasive coronary angiography | 0.81 (0.51 to 1.31) | 0.44 (0.27 to 0.70) | 0.07 |
| Coronary revascularisation | 0.98 (0.69 to 1.38) | 0.75 (0.31 to 1.81) | 0.59 |
| Death or subsequent type 1 or 4b myocardial infarction | 0.76 (0.43 to 1.35) | 0.97 (0.43 to 2.20) | 0.63 |
| * Effect estimates compared between the early computed tomography coronary angiography group and the standard of care only group.  † Models were adjusted for study site (as a random effect), Global Registry of Acute Coronary Events score (using a restricted cubic spline), and prior coronary artery disease.  ‡ Interactions between trial assignment and presentation cardiac troponin were tested. | | | |

**Supplementary Figure 1. Cumulative incidence for overall non-invasive cardiovascular testing.**

Solid lines represent the presence of cardiac troponin elevation; dashed lines represent the absence of cardiac troponin elevation.

Blue lines represent early computed tomography coronary angiography; red lines represent standard of care only.

CI = confidence interval; HR = hazard ratio


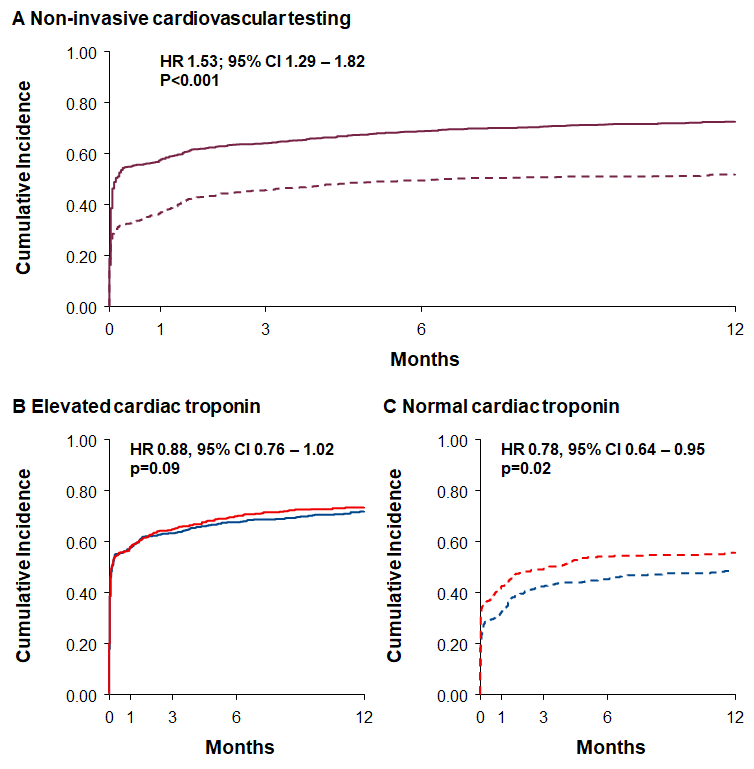


**Supplementary Figure 2. Cumulative incidence for non-invasive cardiovascular testing by category and by cardiac troponin concentration at presentation.**

Solid lines represent the presence of cardiac troponin elevation; dashed lines represent the absence of cardiac troponin elevation.

CI = confidence interval; HR = hazard ratio


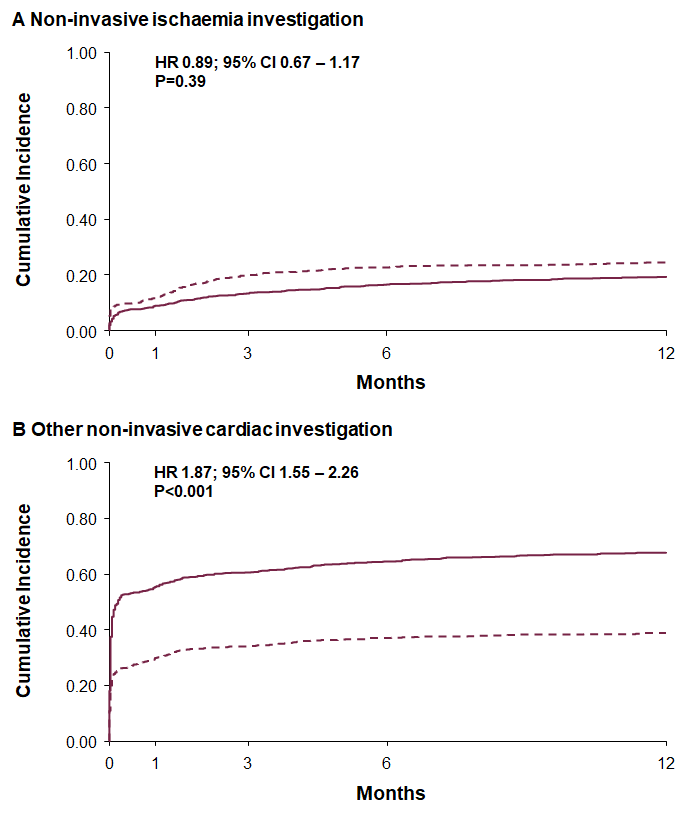


**Supplementary Figure 3. Cumulative incidence for invasive coronary management by cardiac troponin concentration at presentation.**

Solid lines represent the presence of cardiac troponin elevation; dashed lines represent the absence of cardiac troponin elevation.

CI = confidence interval; HR = hazard ratio


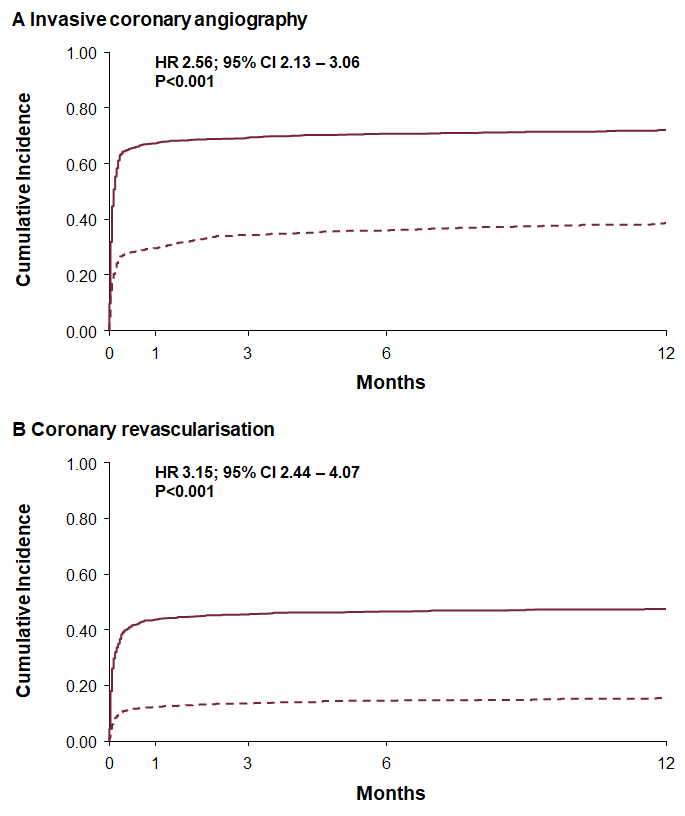


**Supplementary Figure 4. Cumulative incidence for death or subsequent type 1 or 4b myocardial infarction by cardiac troponin concentration at presentation.**

The solid line represents the presence of cardiac troponin elevation; the dashed line represents the absence of cardiac troponin elevation.

CI = confidence interval; HR = hazard ratio


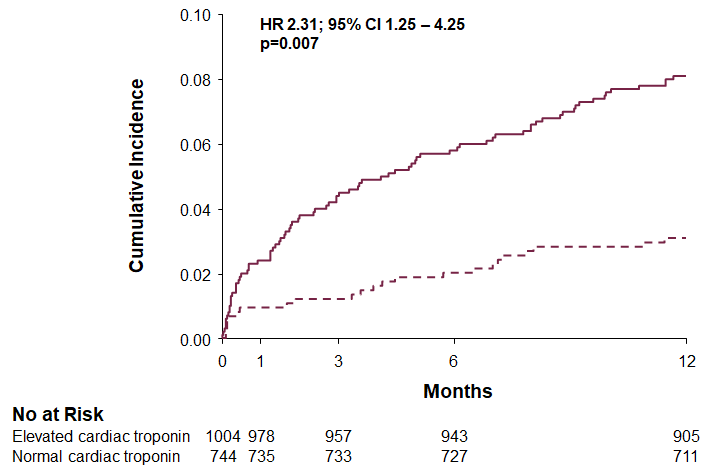

Supplement: zuac057_Supplementary_Data [file zuac057_supplementary_data.docx]
